# Trends and factors associated with the nutritional status of adolescent girls in Ghana: a secondary analysis of the 2003–2014 Ghana demographic and health survey (GDHS) data

**DOI:** 10.1017/S1368980021003827

**Published:** 2022-07

**Authors:** Fusta Azupogo, Abdul-Razak Abizari, Elisabetta Aurino, Aulo Gelli, Saskia JM Osendarp, Hilde Bras, Edith JM Feskens, Inge D Brouwer

**Affiliations:** 1 Division of Human Nutrition and Health, Wageningen University and Research, Wageningen, The Netherlands; 2 Department of Family and Consumer Sciences, Faculty of Agriculture, Food and Consumer Sciences, University for Development Studies, Box TL 1882, Tamale, Ghana; 3 Department of Nutritional Sciences, School of Allied Health Sciences, University for Development Studies, Tamale, Ghana; 4 Centre for Health Economics and Policy Innovation, Department of Management, Imperial College Business School, Imperial College London, London, UK; 5 International Food Policy Research Institute (IFPRI), Washington, DC, USA; 6 Faculty of Arts, The University of Groningen, The Netherlands

**Keywords:** Anaemia, Stunting, Thinness, Overweight, Obesity, Adolescent girls, Ghana

## Abstract

**Objective:**

We examined the trends over time and the factors associated with malnutrition among adolescent girls in Ghana.

**Design:**

Cross-sectional analysis from 3 nationwide Ghana Demographic and Health Surveys conducted in 2003 (*n* 983), 2008 (*n* 955) and 2014 (*n* 857). We used Cox proportional hazard models with sample weighting to model the prevalence ratio (PR) of malnutrition.

**Setting:**

Countrywide, covering rural and urban areas in Ghana.

**Participants:**

Non-pregnant adolescent girls aged 15–19 years.

**Results:**

Compared with 2003, thinness declined marginally (PR 0·88 (95 % CI 0·45, 1·73)) in 2008 and in 2014 (PR 0·71 (95 % CI 0·38, 1·56)). Stunting declined marginally by 19 % in 2008 (PR 0·81 (95 % CI 0·59, 1·12)), flattening out in 2014 (PR 0·81 (95 % CI 0·57, 1·17)). We found an increasing trend of overweight/obesity with the PR peaking in 2014 (PR 1·39 (95 % CI 1·02, 1·88)) compared to 2003. The anaemia prevalence remained severe without a clear trend. A low level of education of the adolescent girl was positively associated with stunting. Increasing age was positively associated with stunting but inversely associated with thinness and anaemia. Girls who ever bore a child were more likely to be anaemic compared to those who never did. A lower level of household wealth and a unit increase in household size was negatively associated with overweight/obesity. Urban dwelling girls were less likely to be stunted.

**Conclusions:**

The stagnant burden of under-nutrition and rising over-nutrition emphasise the need for double-duty actions to tackle malnutrition in all its forms in Ghanaian adolescent girls.

A little over a fifth of the female Ghanaian population is adolescent girls (aged 10–19 years)^(1)^. In addition to physical growth, adolescence is characterised by profound biological, psychosocial and cognitive changes^(2,3)^ related to improved nutrition^(4–6)^. Besides the first 1000 d of life, adolescence offers an additional (and last) critical window of opportunity for linear growth catch-up^(7,8)^.

Nutrient requirements during adolescence are among the highest in the life cycle, making adolescents vulnerable to under-nutrition^(3,9)^ and micronutrient deficiencies, primarily anaemia and iron deficiency anaemia^(10,11)^, while some studies also show an increasing overweight leading to a double burden of malnutrition among adolescents in low- and middle-income countries (LMICs), particularly for girls^(12,13)^. The 2014 Ghana demographic and health survey (GDHS)^(14)^ indicates that 14·0 % of 15–19-year-old female adolescents are thin, and 9·0 % are overweight; other studies show that 44·0 % of rural Ghanaian adolescent girls aged 10–19 years are anaemic, being higher than 60·0 % in the northern and coastal savannah agro-ecological zones^(15)^. This confirms the presence of the double burden of malnutrition among adolescents in Ghana, which has adverse effects on attained height^(16)^, productivity later in life^(17)^ and cardiovascular risk^(18)^.

Malnutrition is also associated with educational, social and economic disadvantages that reduce young people’s capabilities as they mature, contributing to low social and economic status within the household^(19)^. About a third of teenage girls in Ghana are married by the age of 18 years^(1)^, and 14·0 % of those aged 15–19 years have begun childbearing^(14)^, increasing malnutrition risks for themselves and their children^(7,20)^. Girls in Ghana have unhealthier eating habits than boys^(21)^ and are disadvantaged in intra-household food distribution and resource allocation^(22)^. Ghanaian girls are also more likely to drop out of secondary school than boys^(23)^.

The causes of malnutrition are multi-level and can be explained using a conceptual framework, adapted from a recently proposed socio-ecological framework (Fig. 1) for adolescents by our group^(24)^. The framework recognises the complex hierarchical relationship of determinants of nutrition at the environment/community, household and individual level. Individual-level characteristics of the girl such as age, sex, disease, birth order, education, occupation and marital status may affect her nutritional status, mostly through dietary intake, aside from susceptibility and exposure to infection and access to health service^(25)^. Household-level characteristics influence those at the individual level. Some socio-demographic characteristics of girls, such as marital status, may be influenced by parental education and household wealth^(26)^. Household characteristics also influence girls’ empowerment^(27)^, including education, occupation and autonomy; empowerment is an essential determinant of nutrition in many developing contexts^(27–30)^. Place of residence, parental education and occupation and household wealth influence the household’s access to resources, including food, health and sanitation services^(24)^. Poor household access to safe water and sanitation facilities leads to an increased risk of infections and diseases, affecting food intake and utilisation^(31)^. The household’s structure such as a large household size may increase the dependency ratio with consequences for dietary intakes^(32)^ due to competing needs for food and health care. Community- or environmental-level factors are additive factors driving girls’ nutrition directly or through household-level determinants. Cultural and religious norms prevalent in the community influence household behaviours^(33)^. Girls are particularly vulnerable to cultural and gender norms, which often discriminate against them^(34)^.

**Fig. 1:**
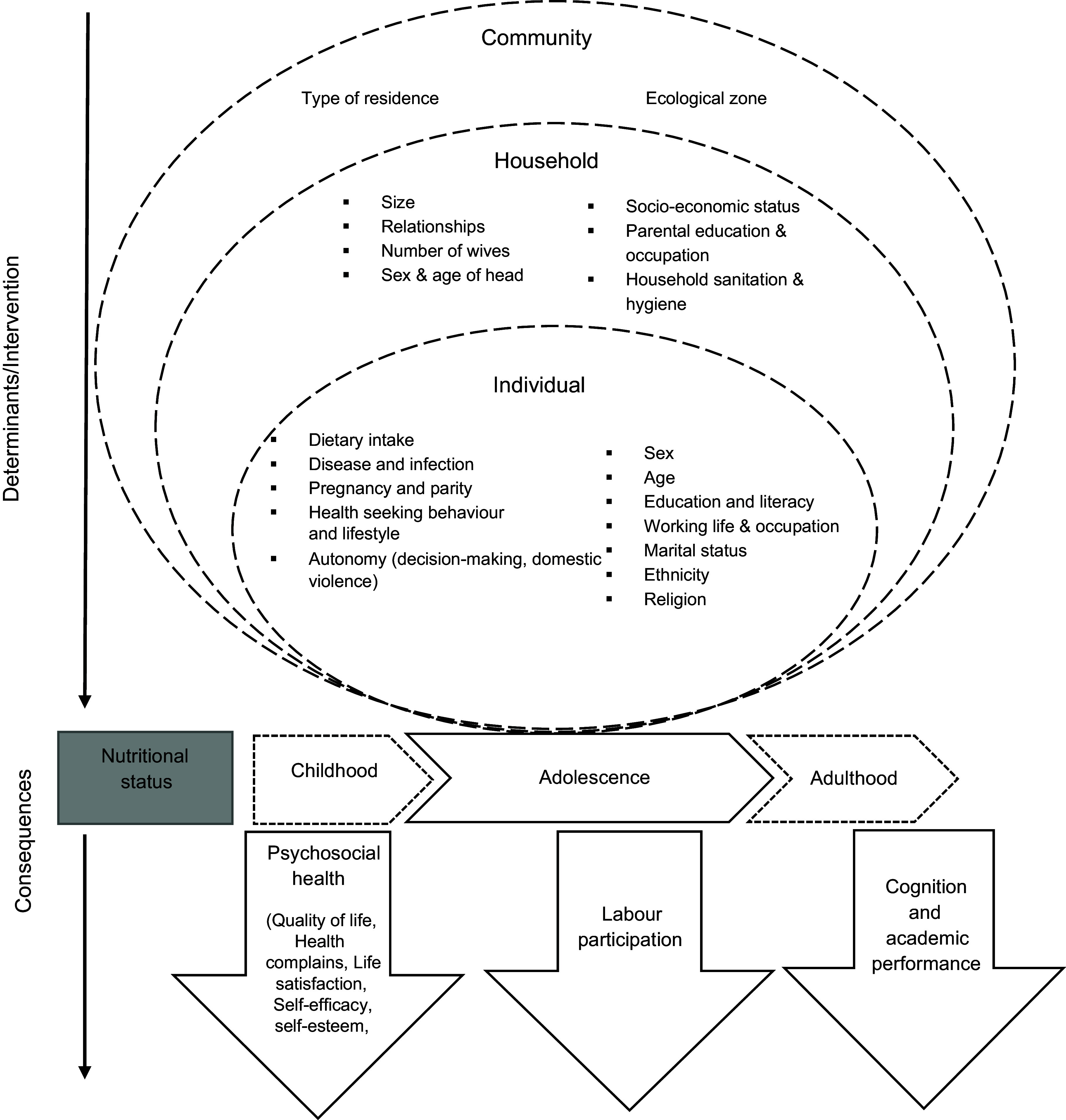
A conceptual framework for the factors associated with the nutritional status of adolescent girls from the 2003–2014 Ghana Demographic and Health Survey. Adapted from Madjdian *et al.* ^(24)^

No national representative data and analysis on determinants of adolescents’ nutrition in Ghana are available. Some studies indicate that dietary intake, parental education and occupation, household socio-economic status, type of residence and ecological zone are predictors of adolescents’ nutrition in Ghana^(11,35,36)^, but the geographic scope and sample size limit the generalizability of these results. Also, no study examined the changes over time in the nutritional status of Ghanaian adolescent girls which may be significant given the massive improvement in socio-economic conditions in the last three decades^(37)^, the second nutrition transition presently occurring in Ghana, mirrored in more imports in the food environment^(38)^ and the consumption of more processed food^(39)^, and the several social protection programmes since the turn of the 21st century, including Livelihood Empowerment Against Poverty Programme and Ghana School Feeding Programme, to reduce poverty and under-nutrition in marginalised and vulnerable groups.

This study aimed to fill this critical knowledge gap by defining the trends over time in the prevalence of malnutrition, including under- and over-nutrition, and the factors associated with malnutrition among adolescent girls in Ghana using nationally representative data included in the GDHS. Our analyses may provide much-desired evidence for policy formulation and programme planning to optimise interventions that optimise nutrition and health for adolescent girls in Ghana.

## Methods

### Study design

We conducted secondary analyses of the national representative 2003, 2008 and 2014 GDHS data for non-pregnant adolescent girls aged 15–19 years. The GDHS contains data on individual demographic characteristics, household characteristics, fertility, women’s empowerment, nutrition and health of Ghanaian women aged 15–49 years. Although available, we did not use the 1993 and 1998 GDHS data due to the absence of Hb data and small sample sizes (see online Supplemental Table S1, for population selected for analysis). Details of the sample selection and data collection of the surveys are presented in the DHS Methodology report^(40)^. The datasets are accessed through the DHS MEASURE website^(41)^. The Ethical Review Committee of Ghana Health Service, Accra, Ghana, approved the GDHS, and no further ethical approval was required. We obtained permission from DHS MEASURE to download and analyse the data.

### Dependent variables

#### Anthropometric indicators

Based on height, weight, age and sex, height-for-age *Z*-score (HAZ) and body-mass-index-for-age *Z*-score (BAZ) were computed with WHO Anthroplus (version 1.0.4), using the WHO 2007 growth reference for 10–19 years adolescent girls. We defined stunting (HAZ < −2 SD), thinness (BAZ < −2 SD), normal weight (−2 SD ≤ BAZ ≤ +1 sd), overweight (+1 SD < BAZ < +2 SD) and obesity (BAZ ≥ +2 SD) in conformity to De Onis *et al.* ^(42)^.

#### Anaemia

In all surveys, Hb concentration was measured with the HemoCue 301, using finger prick by trained health technicians from Ghana Health Service. Hb concentration was adjusted for altitude and smoking. Anaemia and severity were defined using the WHO criteria for non-pregnant girls^(43)^, i.e. Hb < 120 g/l; severe, moderate and mild anaemia as Hb < 80 g/l, 80 ≤ Hb < 110 and 110 ≤ Hb < 120, respectively.

### Independent variables

The following explanatory variables for the girls’ nutritional status were selected based on the conceptual framework and data availability in the GDHS.

#### Individual-level variables

Marriage and fertility-related variables included marital status (categorical), having ever bore a child (dichotomous), and continuous variables for the age at first birth (if any), and the number of children ever born. The girl’s health-seeking behaviour included dichotomous variables of having visited a health facility in the last 12 months, sleeping under a mosquito net and being covered by the national health insurance scheme (NHIS). Lifestyle factors in the analyses included the frequency of watching TV and of listening to the radio in the past week.

The girl’s working life included a dichotomous variable for currently working and a categorical variable for occupation. Girls’ educational status was assessed as a categorical variable and as the number of completed years of schooling. Other demographic characteristics of girls included age in complete years and categorical variables for religion and ethnicity. Data on dietary intake included the frequency of consuming fruits and vegetables in the past week, only available for the 2008 and 2014 surveys and modelled as continuous variables for the survey-specific models. We also included an index of autonomy regarding domestic violence^(44)^ as a proxy of empowerment (see online Supplemental Table S2a); the score ranged from 0 to 5, with a higher score reflecting a greater sense of entitlement and self-esteem and thus higher autonomy^(14)^.

#### Household-level variables

Data included household size, the number of children aged under 5 years, and the household head’s age as continuous predictors; the sex of the household head (dichotomous), the relationship of the girl to household head (categorical) and the socio-economic status of the household defined by the household wealth index (HWI) quintiles. The HWI is a composite measure of a household’s cumulative living standard, calculated using principal components analysis of data on household’s ownership of selected assets, materials used for housing construction, types of water access and sanitation facilities and cooking fuel^(40)^. In the 2008 and 2014 surveys, dichotomous variables for the household ownership of land and farm animals were also included in the analysis. We constructed a composite index of household water and sanitation facilities (WASH) in conformity to the joint WHO/UNICEF guidelines on improved WASH to prevent oral-faecal contamination^(45)^; previous studies^(46,47)^ have used similar indexes (see online Supplemental Table S2b). The WASH index ranged from 0 to 3 based on the available data across surveys.

#### Community and broader environmental-level variables

These included the type of residence and agro-ecological zone. In the GDHS, the countryside was classified as rural residence, while towns and cities were classified as urban^(40)^. The previous ten administrative regions of Ghana used for GDHS were classified into three agro-ecological zones^(48)^, including the: (1) Guinea/Sudan savannah (Northern, Upper East and Upper West Regions); (2) coastal savannah (Central, Greater Accra and Volta Regions) and (3) forest zone (Brong-Ahafo, Ashanti, Western and Eastern Regions) for the analyses.

### Statistical analysis

All statistical analyses were done with SAS 9.4 (SAS Institute Inc.). Statistical significance was considered as a two-tailed *P*-value of ≤0·05 at a 95 % CI. We presented descriptive statistics as percentages for dichotomous/categorical variables and as means (standard errors) for continuous variables. We used trend graphs to map trends over time in mean HAZ, BAZ and Hb and the prevalence of stunting, thinness, overweight/obesity and anaemia. Cox proportional hazard models were fitted to analyse the prevalence ratios (PRs) over time and identify nutritional status determinants over the years with all outcome variables being binary (stunted *v*. not stunted, thin *v*. normal weight, overweight/obese *v*. normal weight and anaemic *v*. not anaemic).

Bivariate analyses were first fitted, and all results with *P*-values ≤0·25 were further assessed in the multivariable models. In the multivariable models, we explored potential interactions by adding pair-wise interaction terms for the determinants, but none was statistically significant. We first created survey-specific models and then pooled the data across all surveys to fit an overall model. In the pooled models, the survey year was included as a categorical variable to examine the trend in the PR with reference to 2003. The log-likelihood ratio test, Akaike information criteria (AIC), Wald test and *P*-value informed the final models. Variables were retained in all final models if they were associated with the outcome variable at a *P*-value of ≤0·05. We applied weighting factors in the data and adjusted for strata and cluster effects using the *PROC SURVEY* function in SAS^(49)^, adjusting for differences in the probability of selection and interview due to the intricate survey design. For the pooled analysis, a combined weighting factor was applied. A detailed explanation of the weighting procedure can be found in the DHS Methodology report^(40)^. In a sensitivity analysis, we repeated all the analyses with linear regression using the ‘*PROC SURVEYREG*’ command in SAS^(49)^ (see online Supplemental Table S4a–c). We further examined the absolute percentage point decrease/increase in malnutrition prevalence between 2003 and 2014 (see online Supplemental Table S5) using SAS ‘*PROC SURVEYREG*’ command^(49)^.

## Results

### Population characteristics

For all surveys (Table 1), the adolescent girls’ mean age was approximately 17 years; about half of the respondents were of Akan ethnicity and more than three-quarters of the adolescents professed Christianity. The majority (≥64·5 %) were unemployed. Most of the girls had secondary/higher education, and the proportion improved marginally from 65·5 % in 2003 to 72·5 % in 2014. Less than 5 % of the girls were wives of the household head. About half resided in rural areas and Ghana’s forest zone. About a quarter of the girls had visited a health facility in the past 12 months. The proportion of girls who slept under a mosquito bed net increased from 4·9 % in 2003 to 28·5 % in 2014. The proportion of those who were currently married decreased from 9·7 % in 2003 to 5·7 % in 2014. About a tenth of the girls ever bore a child with a mean number of births of one child across all years. The score for autonomy improved marginally from 3·7 in 2003 to 4·0 in 2014. The frequency of watching TV decreased from 1·6 in 2003 to 1·2 in 2014 but was highest in 2008 (1·7).

**Table 1:** Population descriptive statistics for adolescent girls from the 2003–2014 Ghana Demographic and health survey data

Variables	Year of survey							
	2003 (*n* 983)		2008 (*n* 955)		2014 (*n* 857)		Pooled data (*n* 2795)	
	Mean or %	se (mean)	Mean or %	se (mean)	Mean or %	se (mean)	Mean or %	se (mean)
Age*	16·9	0·1	17·0	0·1	16·8	0·1	16·9	0·0
Health seeking behaviour and lifestyle								
Visited health facility last 12 months	21·0		25·9		24·9		23·9	
Respondent slept under a mosquito bed net	4·9		16·5		28·5		16·6	
Covered by National Health Insurance (NHIS)	**–**		38·5		56·9		47·7	
Frequency of listening to radio in the past week*	2·0	0·0	2·0	0·1	1·3	0·0	1·8	0·0
Frequency of watching television in the past week*	1·6	0·1	1·7	0·1	1·2	0·0	1·5	0·0
Dietary intake								
Frequency of fruit intake in the past week*	–	–	4·0	0·1	3·0	0·1	3·5	0·1
Frequency of vegetable intake in the past week*	–	–	3·8	0·1	3·3	0·1	3·5	0·1
Demographics of the girl								
Religion								
Christian	82·0		79·9		80·6		80·9	
Muslim	15·4		14·9		17·1		15·8	
Other	2·6		5·1		2·3		3·3	
Ethnicity								
Akan	53·0		50·6		49·3		51·0	
Mole-Dagbani	11·3		15·1		16·6		14·3	
Other	35·7		34·3		34·1		34·7	
Occupation of girl								
Unemployed	65·3		67·7		64·5		65·8	
Agriculture/unskilled labour	21·5		21·2		26·9		23·2	
Skilled labour	13·2		11·1		8·6		11·0	
Girl is currently working	32·0		31·8		33·1		32·3	
Years of schooling*	2·8	0·1	3·0	0·1	3·0	0·1	2·9	0·0
Highest educational level of girl								
No education	11·3		6·9		3·8		7·3	
Primary school	23·2		20·7		23·7		22·5	
Secondary education/higher	65·5		72·4		72·5		70·1	
Marriage, fertility and relations								
Total children ever born*	1·1	0·0	1·2	0·0	1·2	0·1	1·1	0·0
Age at first birth*	16·9	0·1	16·6	0·2	16·6	0·2	16·7	0·1
Girl has ever given birth	10·2		9·8		10·8		10·3	
Marital status								
Never married	88·5		92·9		93·6		61·6	
Formerly married	1·8		0·9		0·7		6·7	
Currently married	9·7		6·1		5·7		31·7	
Relation of girl to the household head								
Household head	2·1		3·9		1·9		2·6	
Wife	4·6		3·7		3·6		4·0	
Daughter	57·6		59·2		62·4		59·8	
Grand-daughter	10·5		9·0		8·0		9·2	
Other family relation	19·2		19·0		15·5		17·9	
Non-family relation	6·0		5·2		8·6		6·5	
Autonomy								
Autonomy index*	3·7	0·1	3·9	0·1	4·0	0·1	3·6	0·0
Household characteristics								
Age of household head*	36·36	1·8	48·7	0·6	49·2	0·7	48·8	0·4
Household size*	6·2	0·1	5·6	0·1	5·7	0·1	5·8	0·1
Number of children < 5 years*	0·8	0·0	0·7	0·0	0·72	0·0	0·7	0·0
WASH index*	2·1	0·0	2·3	0·0	2·3	0·0	2·5	0·0
Sex of household head (male)	57·5		57·7		59·6		58·2	
Household wealth index								
Poorest	14·3		14·5		22·7		17·2	
Poorer	14·2		18·4		22·0		18·2	
Middle	19·1		21·5		18·8		19·8	
Richer	23·7		23·3		17·4		21·5	
Richest	28·7		22·2		19·1		23·3	
Household owns land usable for agriculture	–		49·6		46·1		47·8	
Household owns livestock	–		49·6		48·6		49·1	
Geographical/environmental								
Place of residence								
Rural	55·4		48·8		50·5		51·6	
Urban	44·6		51·2		49·5		48·4	
Agro-ecological zone								
Coastal savannah	35·4		34·6		31·9		34·0	
Forest	51·4		47·7		50·7		49·9	
Guinea/Sudan savannah	13·1		17·7		17·4		16·1	

* Values are means with standard errors, all other values are percentage; Autonomy index, a proxy of autonomy regarding domestic violence; WASH, Household water, hygiene and sanitation.

### The trend in nutritional status and malnutrition

**Fig. 2:**
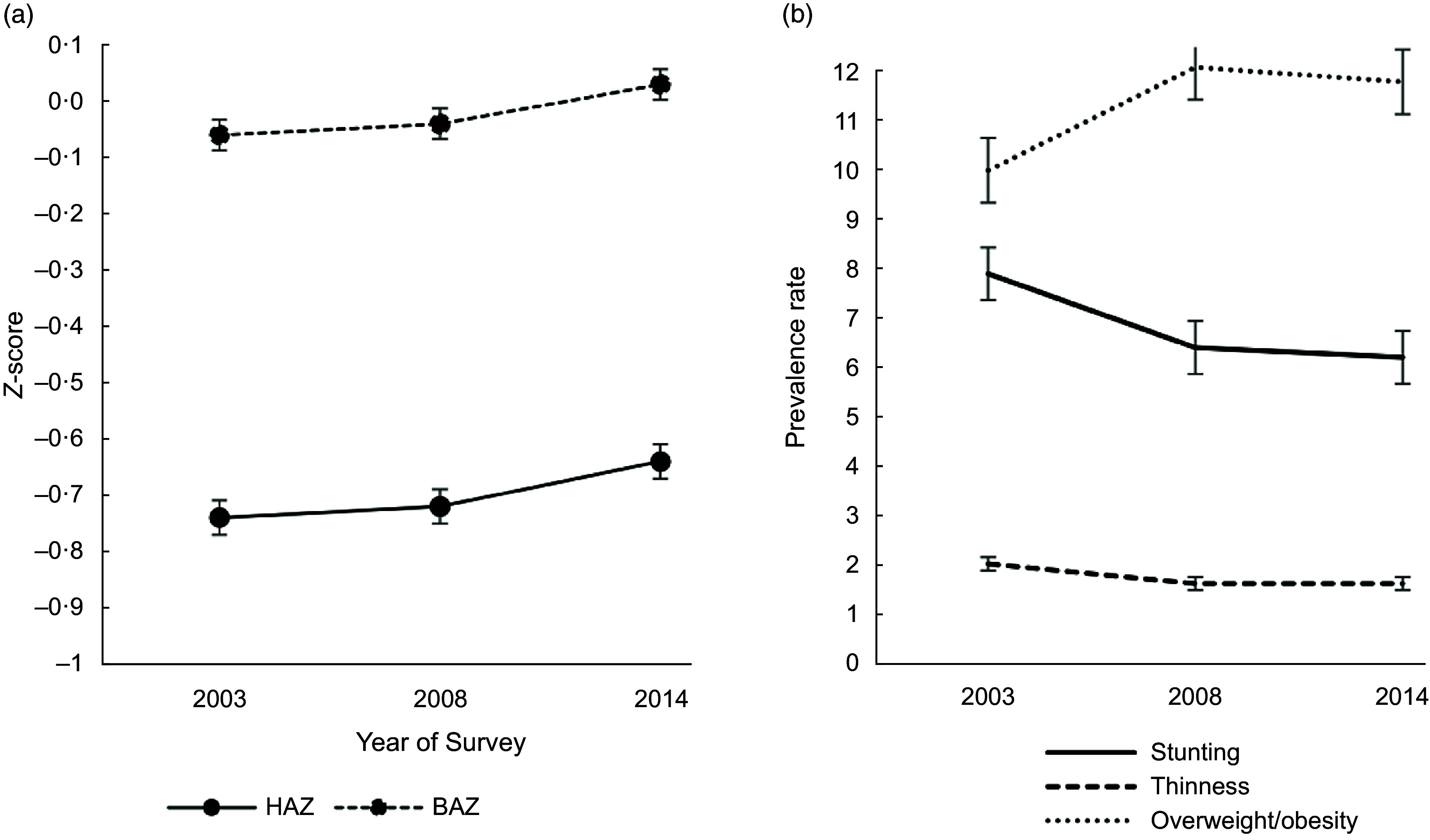
Trend in the: (a) mean height-for-age *Z*-score (HAZ) and mean body-mass-index-for-age *Z*-score (BAZ); (b) prevalence of protein-energy malnutrition among 15–19 years female adolescents from 2003–2014 in Ghana. Vertical bars are standard errors of the (a) arithmetic means and (b) prevalence rates

**Fig. 3:**
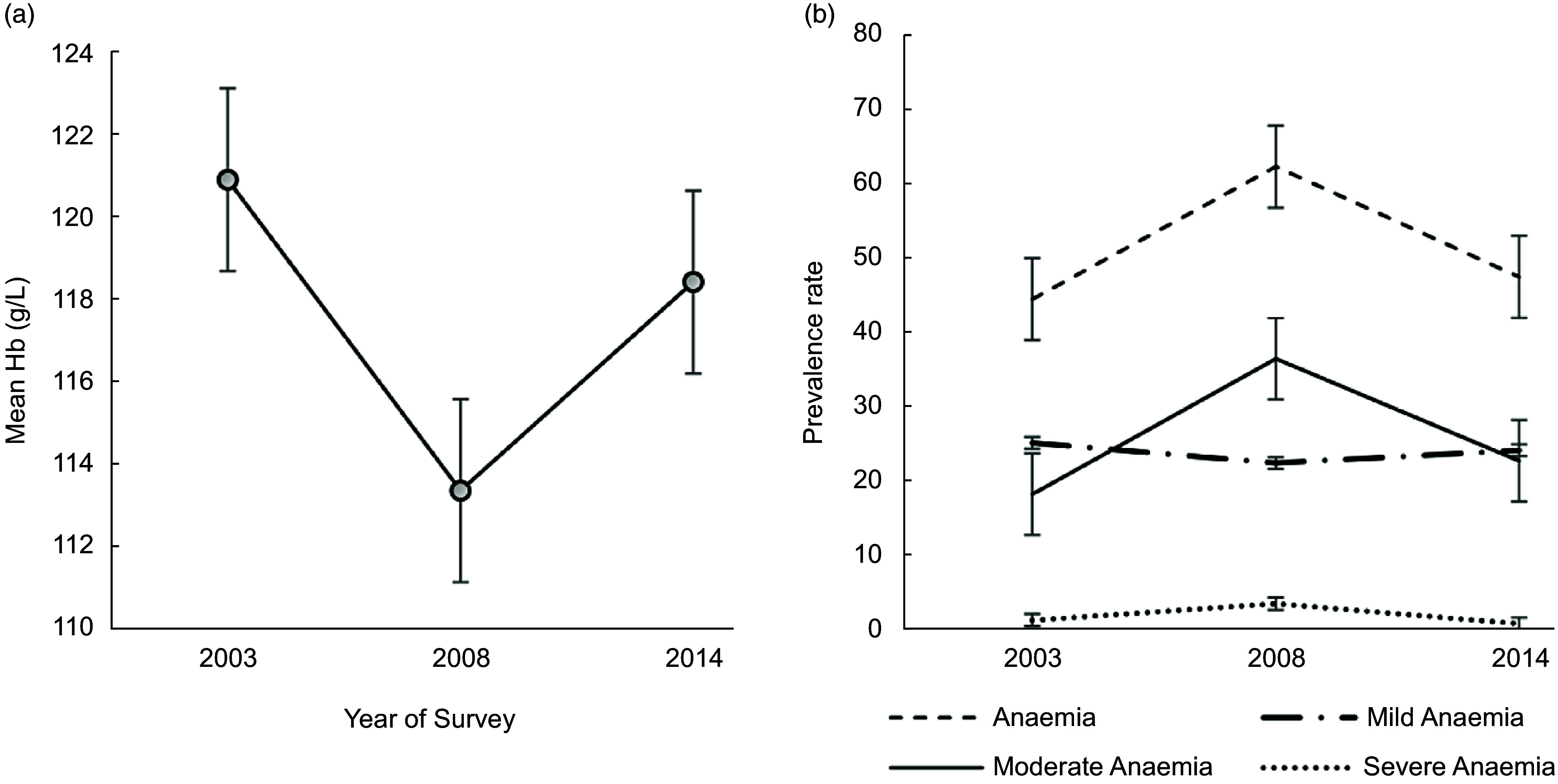
Trend in the: (a) mean Hb (g/l) and (b) anaemia prevalence among female adolescents aged 15–19 years from 2003 to 2014 in Ghana; Hb, haemoglobin; anaemia (Hb <120 g/dl); mild anaemia (110 g/l ≤ Hb ≤ 119 g/l); moderate anaemia (80 g/l ≤ Hb ≤ 109 g/l) and severe anaemia (Hb < 80 g/l). Vertical bars are standard errors of the (a) arithmetic means and (b) prevalence rates

The mean HAZ and BAZ increased non-significantly from 2003 to 2014 (Fig. 2(a)) and the prevalence of stunting and thinness were comparable across the years (Fig. 2(b)). The prevalence of overweight increased from 10·0 % in 2003 to 12·1 % in 2008 but virtually flattened off in 2014 (Fig. 2(b)). We observed a V-shaped curve in the adolescent girls’ mean Hb status between 2003 and 2014 (Fig. 3(a)) with the mean Hb being higher in 2003 (120·9 g/l, se 0·5) compared to 2008 (113·2 g/l, se 0·6) and 2014 (118·4, se 0·6) (Table 2). An inverted V-shape was observed in the prevalence of anaemia between 2003 and 2014, with the 2008 survey recording the highest prevalence of anaemia at 62·1 % (Fig. 3(b)). The prevalence of moderate anaemia changed the most for the surveyed years (Fig. 3(b)). Supplemental Table S3 indicates the prevalence rates of the girls’ nutritional status by year of the survey.

**Table 2:** Multivariate predictors of stunting among non-pregnant adolescent girls: analysis of the 2003–2014 Ghana demographic health survey (GDHS) data

Variables	2003 (*n* 983)		2008 (*n* 955)		2014 (*n* 857)		Pooled (*n* 2795)	
	PPR	95 % CI	PPR	95 % CI	PPR	95 % CI	PPR	95 % CI
Age					1·32	1·11, 1·58**	1·11	1·01, 1·23*
Ethnicity								
Akan	1·73	1·06, 2·83*	0·58	0·32, 1·06				
Mole-Dagbani	0·94	0·39, 2·24	0·47	0·23, 0·96*				
Other (Ref.)	1·00		1·00					
Highest educational level of girl								
No education	1·77	0·87, 3·60			3·30	1·42, 7·65**	1·96	1·29, 2·99**
Primary school	1·99	1·26, 3·15**			1·90	0·95, 3·78	1·93	1·37, 2·72***
Secondary education/Higher (Ref.)	1·00				1·00		1·00	
Autonomy index					0·76	0·63, 0·93**		
WASH index	1·28	1·03, 1·60*	0·67	0·51, 0·88**				
Household wealth index								
Poorest	4·31	1·93, 9·62***						
Poorer	6·80	3·09, 14·95***						
Middle	4·54	2·19, 9·39***						
Richer	4·05	2·07, 7·93***						
Richest (Ref.)	1·00							
Place of residence								
Urban							0·63	0·46, 0·87**
Rural (Ref.)							1·00	
Agro-ecological zone								
Guinea/Sudan savannah	0·60	0·29, 1·23						
Coastal savannah	0·45	0·28, 0·70***						
Forest (Ref.)	1·00							
Survey year								
2003 (ref)							1·00	
2008							0·81	0·59, 1·12
2014							0·81	0·57, 1·17
Model fit statistics								
Wald test		4·55***		6·81***		8·78***		7·25***
−2 Log-likelihood ratio		950·57		780·34		742·42		2924·71
AIC		972·57		786·34		750·42		2936·71

PPR, prevalence risk ratio; 95 % CI, 95 % confidence interval; AIC; Akaike information criteria; N/A, estimates were unreliable (set at zero) as none of the formerly married girls was thin in 2008; Autonomy index, a proxy of autonomy regarding domestic violence; WASH, Household water, hygiene and sanitation.

* *P* ≤ 0 05.

** *P* ≤ 0 01.

*** *P* ≤ 0 001.

**Fig. 4:**
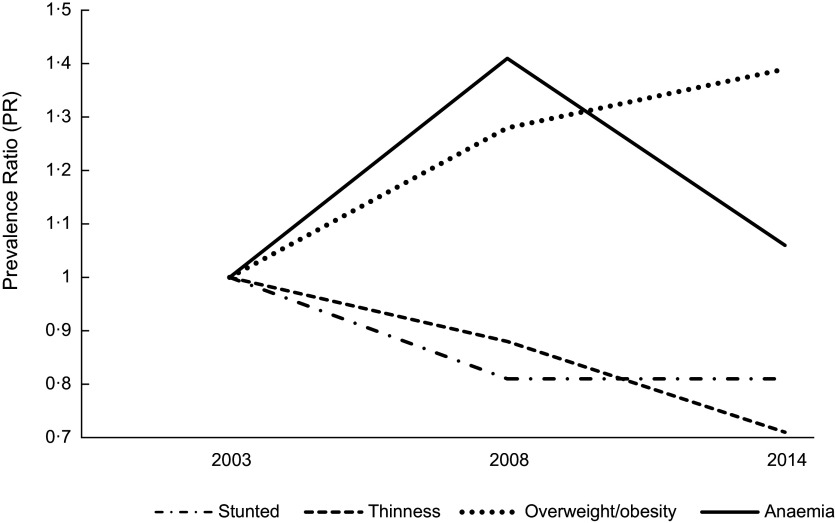
Trend in the adjusted prevalence ratio (PR) of malnutrition among adolescent girls in Ghana from 2003 to 2014; all PRs are adjusted for predictors that were significant in the pooled analysis for each outcome variable

Compared to 2003, the PR of stunting decreased non-significantly by 19 % in 2008 and 2014 respectively (Fig. 4); the PR of thinness declined non-significantly in 2008 compared to 2003, with a further non-significant decrease between 2008 and 2014. Additionally, compared to the 2003 survey, the PR of overweight/obesity increased by 28 % in 2008, peaking significantly at 39 % in 2014. The PR of anaemia increased significantly by 41 % in 2008 compared to 2003, but the trend virtually flattened out in 2014 (Fig. 4).

### Factors associated with the nutritional status of Ghanaian adolescent girls

#### Individual-level factors

Compared to girls with secondary or higher education, those with primary and no education were more likely to be stunted in all but the 2008 survey (Table 2). Girls with no or primary school education were more likely to be thin in 2008 than those with secondary or higher education (Table 3). The PR of anaemia was higher for girls who had ever bore a child in both 2008 and the pooled model (Table 3). In 2014, stunted girls compared to non-stunted girls were more likely to be anaemic (Table 4). Increasing age was positively associated with stunting in 2014 and the pooled model, and with overweight in 2008 (Tables 2 and 5, respectively). Furthermore, increasing age was inversely associated with anaemia in all but 2003 (Table 4). In the 2014 and pooled model, married girls were less likely to be stunted compared to never-married girls (Table 2) and more likely to be overweight/obese compared to never-married girls in 2014 and the pooled model (Table 5). The association between ethnicity and stunting was inconclusive, with conflicting results in 2003 and 2008. However, girls from the Akan ethnicity were significantly less likely to be overweight or obese in both 2008 and the pooled analysis. A unit increase in the autonomy index was inversely associated with stunting (Table 2) and positively associated with overweight/obesity in 2014 (Table 5). A unit increase in the frequency of watching TV was inversely associated with thinness in the pooled model (Table 3) and positively associated with overweight/obesity in 2003 (Table 5). For a unit increase in the frequency of listening to the radio, the PR of thinness increased by 48 % in 2003 (Table 3). A unit increase in fruit consumption frequency significantly reduced the thinness PR in 2014 (Table 3).

**Table 3:** Multivariate predictors of thinness among non-pregnant adolescent girls: analysis of the 2003–2014 Ghana demographic health survey (GDHS) data

Variables	2003 (*n* 983)		2008 (*n* 955)		2014 (*n* 857)		Pooled (*n* 2795)	
	PPR	95 % CI	PPR	95 % CI	PPR	95 % CI	PPR	95 % CI
Age							0·75	0·56, 0·99*
Frequency of listening to the radio in the past week	1·46	1·09, 1·96*						
Frequency of watching television in the past week	0·61	0·38, 0·98*					0·68	0·51, 0·91**
Frequency of fruit intake in the past week					0·76	0·58, 0·99*		
Highest educational level of girl								
No education	1·79	0·52, 6·11	1·25	0·15, 10·57				
Primary school	2·70	1·02, 7·11*	3·53	1·16, 10·73*				
Secondary education/Higher (Ref.)	1·00		1·00					
Survey year								
2003 (ref)							1·00	
2008							0·88	0·45, 1·73
2014							0·71	0·38, 1·56
Model fit statistics								
Wald test	4·81***			3·59*		3·74*		2·90*
−2 Log-likelihood ratio	228·02			185·55		190·66		711·36
AIC	236·02			189·55		192·66		719·36

PPR, prevalence risk ratio; 95 % CI, 95 % confidence interval; AIC, Akaike information criteria.

* *P* ≤ 0·05.

** *P* ≤ 0·01.

*** *P* ≤ 0·001.

**Table 4:** Multivariate predictors of anaemia among non-pregnant adolescent girls: analysis of the 2003–2014 Ghana demographic health survey (GDHS) data

Variables	2003 (*n* 983)		2008 (*n* 955)		2014 (*n* 857)		Pooled (*n* 2795)	
	PPR	95 % CI	PPR	95 % CI	PPR	95 % CI	PPR	95 % CI
Age			0·94	0·91, 0·98**	0·88	0·83, 0·94***	0·94	0·91, 0·97***
Girl has ever given birth								
Yes	1·25	1·03, 1·51*	1·21	1·00, 1·45*			1·22	1·07, 1·40**
No (Ref.)	1·00		1·00				1·00	
Stunting status								
Yes					1·48	1·12, 1·98***		
No (Ref.)					1·00			
Relation of girl to the household head								
Household head			0·87	0·61, 1·23				
Wife			1·07	0·81, 1·40				
Grand-daughter			1·01	0·85, 1·21				
Other family relation			0·92	0·79, 1·07				
Non-family relation			0·59	0·39, 0·89**				
Daughter (Ref.)			1·00					
Household owns land								
Yes					1·27	1·07, 1·50**		
No (Ref.)					1·00			
Agro-ecological zone								
Guinea/Sudan savannah			0·81	0·71, 0·93*	0·96	0·80, 1·16		
Coastal savannah			0·81	0·71, 0·93*	1·23	1·01, 1·50*		
Forest (Ref.)			1·00		1·00			
Year of survey								
2003 (Ref)							1·00	
2008							1·41	1·28, 1·56***
2014							1·06	0·95, 1·19
Model fit statistics								
Wald test	5·27*			3·99***		6·77***		19·30***
−2 Log-likelihood ratio	5187·97			7629·05		5787·79		21 614·57
AIC	5189·97			7647·05		5797·79		21 622·57

PPR, prevalence risk ratio; 95 % CI, 95 % confidence interval; AIC; Akaike information criteria.

* *P* ≤ 0·05.

** *P* ≤ 0·01.

*** *P* ≤ 0·001.

**Table 5:** Multivariate predictors of overweight/obesity among non-pregnant adolescent girls: analysis of the 2003–2014 Ghana demographic health survey (GDHS) data

Variables	2003 (*n* 983)		2008 (*n* 955)		2014 (*n* 857)		Pooled (*n* 2795)	
	PPR	95 % CI	PPR	95 % CI	PPR	95 % CI	PPR	95 % CI
Age			1·15	1·01, 1·30*				
Frequency of watching television in the past week	1·29	1·09, 1·53**						
Autonomy					1·27	1·00, 1·62*		
Ethnicity								
Akan			0·62	0·41, 0·93*			0·71	0·56, 0·90**
Mole-Dagbani			0·82	0·49, 1·40			0·82	0·56, 1·20
Other (Ref.)			1·00		1·00		1·00	
Marital status								
Currently married					3·52	1·84, 6·75***	1·03	0·65, 1·63
Formerly married					0·67	0·23, 1·91	2·54	1·03, 6·26*
Never married (Ref.)					1·00		1·00	
Household size	0·91	0·86, 0·97**	0·88	0·81, 0·95***			0·93	0·89, 0·98**
Household wealth index								
Poorest	0·15	0·04, 0·64***	0·55	0·29, 1·05	0·21	0·10, 0·41***	0·24	0·15, 0·39***
Poorer	0·18	0·08, 0·40***	0·53	0·31, 0·92*	0·15	0·07, 0·35***	0·23	0·15, 0·35***
Middle	0·24	0·11, 0·55***	0·33	0·17, 0·63***	0·51	0·28, 0·93*	0·34	0·23, 0·49***
Richer	0·70	0·46, 1·05	0·86	0·56, 1·31	0·79	0·48, 1·29	0·76	0·59, 0·98*
Richest (Ref.)	1·00		1·00		1·00		1·00	
Year of survey								
2003 (Ref)							1·00	
2008							1·28	0·98, 1·67
2014							1·39	1·02, 1·88*
Model fit statistics								
Wald test		11·74***		6·01***		9·10***		11·33***
−2 Log-likelihood ratio		1178·16		1472·42		1400·53		4769·52
AIC		1190·16		1488·42		1414·53		4791·52

PPR, prevalence risk ratio; 95 % CI, 95 % confidence interval; AIC; Akaike information criteria; autonomy index, a proxy of autonomy regarding domestic violence.

* *P* ≤ 0 05.

** *P* ≤ 0.01.

*** *P* ≤ 0 001.

#### Household-level factors

A unit increase in the WASH index was positively associated with stunting in 2003 but inversely associated with stunting in 2008 (Table 2). A lower HWI was positively associated with stunting for only the 2003 survey (Table 2). Girls in the first four quintiles of the HWI compared to the fifth quintile were less likely to be overweight or obese for all survey years and the pooled analysis (Table 5). Except for the 2014 survey, a unit increase in household size was inversely associated with overweight/obesity (Table 5). Household land ownership was significantly associated with anaemia in 2014 (Table 4). Compared to girls that were daughters of the household head, the PR of anaemia was significantly lower for girls who were not related to the household head (Table 4).

#### Community-level factors

In our pooled analysis, urban girls were significantly less likely to be stunted than their rural peers (Table 2). Furthermore, compared to girls who resided in Ghana’s forest zone, those who resided in the coastal savannah zone were significantly more likely to be stunted in 2003 (Table 2) and those residing in the coastal and Guinea/Sudan savannah zones were less likely to be anaemic in 2008 (Table 4). However, girls in the coastal savannah zone were significantly more likely to be anaemic in 2014 (Table 4).

#### Model fit and sensitivity analysis

The model fit statistics showed that all the models were statistically significant. Our sensitivity analysis (see online Supplemental Table S4a–c) showed a similar trend for HAZ, BAZ, Hb and the factors associated with each of these outcomes. Lower HWI quintiles were significantly associated with a lower HAZ and BAZ in all the statistical models. Being thin was associated with a lower Hb in all but the 2014 survey model. Between 2003 and 2014, stunting decreased slightly by only 1·5 % points and underweight by less than 1 %-point difference but, overweight increased significantly by 4·3 % (95 % CI 0·74, 7·84) points for the adolescent girls (see online Supplemental Table S5). Anaemia increased significantly by 18·1 % points in 2008 with a minor increase 2·82 % (95 % CI -1·76, 7·41) in 2014 compared to 2003.

## Discussion

This study examined the trends over time and the factors associated with malnutrition among adolescent girls aged 15–19 years in Ghana using nationally representative data from the 2003, 2008 and 2014 GDHS. According to the WHO criterion, the prevalence of anaemia in all the surveys was of severe public health significance^(43)^, confirming previous studies^(15,50)^. The severity of anaemia over the years suggests a high burden of micronutrient deficiencies among adolescent girls in Ghana; inadequate dietary intake, evolving from food insecurity and the consumption of monotonous plant-based diets with little or no animal source foods, has been cited as a common underlying cause^(9,51)^.

The 2008 survey coincided with the global financial crisis during which macro-economic growth in Ghana was marginal compared to the previous years, with spikes in the prices of fuel and food^(52)^. Household food and non-food expenditure are associated with household dietary diversity^(53)^. Hence, the spike in fuel and food prices plausibly influenced household food security and diversity negatively, especially among the middle-class and poor without adequate safety nets. The preceding may partly account for the peak in anaemia in 2008. Besides the effects of the global financial crisis, the finding may also relate to the prevalence and type of disease vectors during the survey. Our analysis showed that the peak of anaemia in 2008 was highest in the forest zone (*not shown*), which was contrary to other studies in Ghana^(15,54)^. The forest zone of Ghana has a tropical climate in which malaria exposure is higher^(55)^; although we could not verify this with the available data, a recent study found that while anaemia in children and reproductive women was associated with iron deficiency in northern Ghana (Guinea savannah zone), it was rather associated with inflammation in the middle and southern belts (forest zone) of the country^(56)^.

Stunting and underweight declined non-significantly between 2003 and 2014 for the adolescent girls, corroborating the finding of Black *et al.* ^(57)^, who report that, globally, stunting is decreasing slowly. Though Ghana attained middle-income status in 2005, inequality has been increasing, and poverty remains prevalent in many areas, with increasing urban poverty resulting from high graduate unemployment^(58)^. Food security plays a significant role in the prevalence of thinness among adolescents in LMICs^(59)^. Ghana was among the first African countries to achieve the first Millennium Development Goal of ‘*eradicating extreme poverty and hunger*’. However, a heavy reliance on rain-fed agriculture, inflation and high food prices continuously pose a threat to food security even in urban Ghana^(60)^, partly accounting for the stagnant burden of stunting and minor decrease in thinness. In reality, declines in stunting are only noticeable after a couple of generations of better-nourished mothers^(57)^; but the 11-year trend in our study sufficed to observe a trend.

Together with the stagnating under-nutrition rates, we observed an increasing trend in adolescent overweight/obesity over the years. Although our study is the first to map the trend over time in Ghanaian adolescent girls’ nutritional status, Ofori-Asenso and colleagues^(61)^ observed an increasing trend in overweight and obesity prevalence for Ghanaian adults in the period 1998–2016 with women more overweight and obese in their study. Increases in overweight and obesity can happen more rapidly than declines in (chronic) under-nutrition^(12,13)^, leading to the co-existence of over- and under-nutrition. Ghana is in the second phase of the nutrition transition^(39)^ with increasing consumption of processed foods, ‘*fast-foods*’ and energy-dense snacks alongside decreasing physical activity levels, which have contributed to overweight and obesity^(11,62,63)^. Buxton^(64)^ found that adolescents in Ghana have unhealthy eating patterns and habits, which are worst among adolescent girls^(21)^ and may partially explain our findings. Also, adolescent girls in Ghana are known to have less physical activity than their male peers^(65)^. Overall, the co-existence of under-nutrition and over-nutrition is a reflection of persistent food insecurity and poverty alongside a nutrition transition with an increasingly sedentary lifestyle^(63)^.

Similar to the WHO report^(25)^, early child-bearing and socio-economic factors (education, household wealth, type of residence) significantly predicted the nutritional status of the adolescent girls in our study. In detail, individual-level characteristics associated with the girls’ malnutrition included: (1) age; (2) education and (3) whether the girl ever bore a child. First, educational status is a proxy of socio-economic status and empowerment^(66)^; accordingly, higher educated girls may be more empowered and less impoverished. Better education may protect against adverse nutrition and health outcomes through the acquisition of positive social, psychological and economic skills and by influencing lifestyle behaviours such as healthy food choices^(67)^. Less-educated adolescents are likely to be from households with low socio-economic status^(24,68)^, associated with a lower HAZ in the present study.

Second, in contrast to our previous study^(15)^, increasing age seemed the most reliable determinant of a reduced anaemia prevalence. Girls in our sample were in fertile age compared to our previous study, where the girls were primarily pre-menarche. Also, other studies found increasing age to be protective of anaemia^(69,70)^. One possible reason is that older girls may be less susceptible to chronic infection and inflammation^(71)^. Many studies have reported younger age as a risk factor for stunting among children and adolescents in LMICs^(72,73)^, but our study shows that older girls are more stunted. Although this finding conforms with Leslie and Pawloski^(74)^, it was unexpected and does not support the evidence of catch-up growth or compensatory gain among adolescents. Catch-up growth among adolescents may occur only if there is a significant maturational delay of 1 to 2 years to allow additional growth^(75)^.

Lastly, girls who had borne a child before were more likely to be anaemic in our analysis. Pregnancy poses an extra-demand of nutrient requirements for the growing foetus^(3,9,34)^. Adolescent pregnancy negatively affects the girl’s linear growth, increasing their risk for stunting^(20)^. Stunted children and adolescents are more susceptible to chronic infections and inflammation^(25)^, this predisposes them to micronutrient deficiencies, including anaemia. Reduced linear growth is also associated with intergenerational consequences of adverse birth outcomes^(20)^. Our data suggest marriage could influence the association of childbearing and nutrition status as married girls were older and more likely to have borne a child. Girls who mature early look older and marry earlier, partly attributed to better secondary sex characteristics for heavier girls^(76)^. In reality, the socio-economic and physiologic deprivations associated with teenage marriage^(9,20,34,77)^ outweigh any possible benefits in the girl’s nutrition and health. Moreover, any possible benefits of teenage marriage largely depend on the partner’s socio-economic status.

Our results suggest that while higher autonomy has benefits for stunting reduction, it is also positively associated with being overweight. Adolescent girls who are more autonomous may have more control over household resources and are better able to make independent decisions regarding their health, including reproductive health^(44)^; this probably explains the negative association between increasing autonomy index and stunting in 2014. Equally, girls with a higher autonomy may have more purchasing power, which may probably result in more consumption of ‘*fast-foods*’ and energy-dense snacks, explaining the positive trend between the autonomy index and overweight/obesity in 2014.

We observed a positive trend between the frequency of TV watching and overweight/obesity in 2003, with an inverse trend for thinness in our pooled analysis. A combination of the frequency and amount of time spent watching TV or listening to the radio would be a better measure of a sedentary lifestyle^(78)^, but these data were not available. Previous studies showed that a higher frequency of TV watching is significantly associated with overweight for adolescents^(68)^ and women aged 15–49 years^(79,80)^. Children who frequently watch TV are also more likely to consume energy-dense snacks and sugar-sweetened beverages^(68)^, which contributes to a higher energy intake, increasing the likelihood of overweight/obesity.

The most consistent household determinant of the adolescent girls’ malnutrition was the household size and HWI. Household size was inversely associated with overweight/obesity in our study. A higher dependency ratio may increase household expenditures and competing household needs may lower dietary quantity and quality, with consequences for weight loss, micronutrient deficiencies, infections and stunting^(51)^. Girls from households in the first four lower HWI quintiles were consistently less likely to be overweight/obese but were only more likely to be stunted in 2003. In our sensitivity analysis with linear regression, lower levels of HWI were negatively associated with HAZ and BAZ, suggesting that increasing household wealth may significantly increase overweight/obesity with a marginal reduction in stunting. One probable reason is that a short- to medium-term exposure to improved household wealth may rapidly improve dietary intake and health; this would improve weight in the short to medium term. Also, girls from deprived households may lack the purchasing power to consume ‘*fast-foods*’ and energy-dense snacks, which may reduce overweight/obesity. In contrast, a long-term exposure to improved household wealth would be desirable in reducing stunting^(25,57)^. Overall, improving socio-economic conditions is a well-known determinant of a reduced risk of under-nutrition but an increased risk of over-nutrition^(11,24,35,73)^.

Households with access to agricultural land are more likely to have improved livelihoods^(81)^, especially in rural communities with farm-based livelihoods. Nevertheless, girls from such settings may also be overburdened with farm-related work alongside their gender roles of household chores, compromising their health. The above may explain that girls from households with land were more likely to be anaemic in the 2014 survey. Undeniably, women in Ghana are known to have more substantial burdens in their time than men^(82)^, and adolescent girls may be no exception. The work burden may lead to stress with probable consequences for poor dietary and health-seeking behaviours, impacting health negatively. For instance, the risk of micronutrient deficiencies was reportedly higher among working than non-working girls in Sri Lanka^(83)^. Also, children who worked longer hours were allegedly more stunted than their peers who worked for shorter hours in Nepal^(84)^.

Community-level determinants of malnutrition for the girls included the agro-ecological zone and the type of residence. However, the type of residence was only a significant determinant of stunting in our pooled analysis, and the association between agro-ecological zone and malnutrition was inconsistent. Overall, poverty and food insecurity are more prevalent in rural parts of the country than in urban settings^(58,60)^; this may partly explain that urban-dwelling girls were less stunted. Moreover, many rural communities still have poor access to sanitation services and health care, despite introducing the Community-Based Health Planning and Services compounds in rural Ghana in the early 2000s. Contrary to previous studies^(15,54)^, girls residing in Ghana’s coastal and Guinea/Sudan savannah zones compared to the forest zone were remarkably less likely to be anaemic in the 2008 survey; as earlier mentioned, this partly explains why the prevalence of anaemia peaked in 2008 and somewhat relates to the 2008 global financial crisis and the type and prevalence of disease vectors.

### Policy implications

Our findings emphasise the importance of double-duty actions proposed by Hawkes and colleagues^(85)^ to tackle both under- and over-nutrition, but evidence of effectiveness for adolescent girls remains unclear. Until recently, nutrition initiatives in Ghana commonly focused on infants, young children and women, neglecting adolescents. The few interventions targeting adolescents lately have mainly concentrated on improved micronutrient intake for adolescent girls and reduced schistosomiasis and soil-transmitted helminths among school children. The ‘Girls, Iron-Folate Tablet Supplementation (GIFTS)’ programme for junior high school girls in Ghana^(86)^ may help reduce anaemia, although compliance-related issues^(87,88)^ may limit its effectiveness. While the school provides a reliable platform for in-school girls, innovative programmes targeting out-of-school girls are also desirable.

Considering that girls in rural settings were more likely to be stunted, there is a continued need for policies that enhance food security in low-income communities and households and improve girl-child education to mitigate the flattening stunting rate. Likewise, nutrition and public health policies should target girls in high socio-economic settings to overcome the increasing over-nutrition trend. Such programmes may include sensitisations and education to improve the consumption of healthier snacks such as fruits and promote a healthy and active lifestyle during adolescence, such as aerobic outdoor games.

The burden of anaemia emphasises a need for a multi-sectoral approach to anaemia prevention. Only about a quarter of the girls ever visited a health facility in the last 12 months; while this may suggest respondents were generally healthy, it also underscores the need to promote health-seeking behaviour among adolescent girls. Our study shows that teenage pregnancy and teenage marriage is still prevalent and efforts to prevent these should be strengthened including improved reproductive health education and care.

### Limitations

The present study is the first to map the trend in malnutrition among adolescent girls in Ghana and to assess the factors associated with this malnutrition using national representative data. Nonetheless, our analysis is not without challenges. Firstly, it was impossible to model some potential explanatory variables in the pooled analysis since the data were not available in all datasets. Secondly, dietary intake data were limited to fruits and vegetable consumption, and the data did not include household food security although being an important determinant of adolescent nutritional status^(89)^. Thirdly, menstruation increases the risk of micronutrient deficiencies, notably, iron deficiency anaemia through iron loss in the blood^(9)^. However, we could not include menarche status in our analysis. Data on menstruation were related to whether or not the girl menstruated in the last 6 weeks preceding the survey, but we are not certain whether girls who did not menstruate 6 weeks before the surveys were pre-menarche or simply missed their menstrual period. Fourthly, we selected a subset of women in fertile age (15–49 years) and were not able to include data for 10–14-year-old adolescents since they are not part of the fertile age group. We were, therefore, limited in examining the trend and correlates of malnutrition for only older adolescents aged 15–19 years. Our findings may, therefore, be extrapolated only to 15–19 years adolescent girls in Ghana. Finally, the GDHS surveys used a cross-sectional study design, and our findings only depict associations.

## Conclusions

Our findings point to a stagnant burden of under-nutrition with an existing and upcoming burden of over-nutrition for non-pregnant adolescent girls in Ghana. Nutrition interventions should consider adolescent girls as a major target group besides the usual priority groups, infants and young children. Our findings emphasise the need for double-duty actions to tackle both under- and over-nutrition holistically. Different intervention programmes are needed to meet the nutrition-specific needs of different socio-economic groups of adolescent girls.

## Supplementary material

For supplementary material accompanying this paper visit https://doi.org/10.1017/S1368980021003827. click here to view supplementary material

## References

[1] Ghana Statistical Service (2013) 2010 Population and Housing Census Report. Children, Adolescents & Young People in Ghana. Accra, Ghana: Ghana Statistical Service.

[2] Schroeder K & Sonneville K (2016) Adolescent nutrition. In Encyclopedia of Food and Health, pp. 43–50 [ B Caballero , PM Finglas and F Toldrá , editors]. Burlington, UK: Academic Press.

[3] Stang J & Story M (editors) (2005) Guidelines for Adolescent Nutrition Services. Minneapolis, MN: Center for Leadership, Education, and Training in Maternal and Child Nutrition, Division of Epidemiology and Community Health, School of Public Health, University of Minnesota.

[4] Dercon S & Sánchez A (2013) Height in mid childhood and psychosocial competencies in late childhood: evidence from four developing countries. Econ Hum Biol 11, 426–432. 23669463 10.1016/j.ehb.2013.04.001

[5] Crookston R , McClellan C , Georgiadis A et al. (2014) Factors associated with cognitive achievement in late childhood and adolescence: the young lives cohort study of children in Ethiopia, India, Peru, and Vietnam. BMC Pediatr 14, 1–9. 25282338 10.1186/1471-2431-14-253 PMC4193389

[6] Chiplonkar SA & Kawade R (2014) Linkages of biomarkers of zinc with cognitive performance and taste acuity in adolescent girls. Int J Food Sci Nutr 65, 399–403. 24490852 10.3109/09637486.2014.880667

[7] Prentice AM , Ward KA , Goldberg GR et al. (2013) Critical windows for nutritional interventions against stunting. Am J Clin Nutr 97, 911–918. 23553163 10.3945/ajcn.112.052332 PMC3628381

[8] Thurnham DI (2013) Nutrition of adolescent girls in low- and middle-income countries. Sight Life 27, 26–37.

[9] Mesías M , Seiquer I & Navarro MP (2013) Iron nutrition in adolescence. Crit Rev Food Sci Nutr 53, 1226–1237. 24007425 10.1080/10408398.2011.564333

[10] Korkalo L , Freese R , Alfthan G et al. (2015) Poor micronutrient intake and status is a public health problem among adolescent Mozambican girls. Nutr Res 35, 664–673. 26077868 10.1016/j.nutres.2015.05.013

[11] Alicke M , Boakye-Appiah JK , Abdul-Jalil I et al. (2017) Adolescent health in rural Ghana: a cross-sectional study on the co-occurrence of infectious diseases, malnutrition and cardio-metabolic risk factors. PLoS One 12, 1–15. 10.1371/journal.pone.0180436 PMC5519039 28727775

[12] Jaacks LM , Slining MM & Popkin BM (2015) Recent trends in the prevalence of under and overweight among adolescent girls in low- and middle-income countries. Pediatr Obes 10, 428–435. 25558987 10.1111/ijpo.12000 PMC4492920

[13] Caleyachetty R , Thomas GN , Kengne AP et al. (2018) The double burden of malnutrition among adolescents: analysis of data from the global school-based student health and health behavior in school-aged children surveys in 57 low- and middle-income countries. Am J Clin Nutr 108, 414–424. 29947727 10.1093/ajcn/nqy105

[14] Ghana Statistical Service, Ghana Health Service & ICF International (2015) Demographic and Health Survey 2014. Accra, Ghana and Rockville, Maryland, USA: Ghana Statistical Service.

[15] Azupogo F , Aurino E , Gelli A et al. (2018) Agro-ecological zone and farm diversity are factors associated with haemoglobin and anaemia among rural school-aged children and adolescents in Ghana. Matern Child Nutr 15, 1–11. 10.1111/mcn.12643 PMC7198936 30047257

[16] Fink G & Rockers PC (2014) Childhood growth, schooling, and cognitive development: further evidence from the young lives study. Am J Clin Nutr 100, 182–188. 24808488 10.3945/ajcn.113.080960

[17] Das JK , Lassi ZS , Hoodbhoy Z et al. (2018) Nutrition for the next generation: older children and adolescents. Ann Nutr Metab 72, Suppl. 3, 49–57. 10.1159/000487385 29631269

[18] Anyaegbu E & Dharnidharka V (2015) Hypertension in the teenager. Pediatr Clin North Am 61, 131–151. 10.1016/j.pcl.2013.09.011 PMC3947917 24267462

[19] Hardgrove A , Pells K , Boyden J et al. (2014) Youth Vulnerabilities in Life Course Transitions. 2014 UNDP Human Development Report Office. Occasional Paper 3. New York, USA: UNDP.

[20] Kawakita T , Wilson K , Grantz KL et al. (2015) Adverse maternal and neonatal outcomes in adolescent pregnancy. J Pediatr Adolesc Gynecol 29, 130–136. 26327561 10.1016/j.jpag.2015.08.006 PMC4886236

[21] Amos PM , Intiful FD & Boateng L (2012) Factors that were found to influence Ghanaian adolescents’ eating habits. SAGE Open 2, 1–6.

[22] Doss C (2013) Intrahousehold Bargaining and Resource Allocation in Developing Countries. Policy Research Working Paper 6337. Washington, DC: The World Bank.

[23] FAO (2012) Gender Inequalities in Rural Employment in Ghana: An Overview. Rome, Italy: FAO.

[24] Madjdian DS , Azupogo F , Osendarp S et al. (2018) Socio-cultural and economic determinants and consequences of adolescent undernutrition and micronutrient deficiencies in LLMICs: a systematic narrative review. Ann N Y Acad Sci 1416, 117–139.

[25] WHO (2005) Nutrition in Adolescence: Issues and Challenges for the Health Sector. Issues in Adolescent Health and Development. WHO Discussion Papers on Adolescence. Geneva, Switzerland: WHO.

[26] UNICEF (2019) Adolescent Girls’ Health and Well-Being in West and Central Africa. Geneva, Switzerland: UNICEF.

[27] Amugsi DA , Lartey A , Kimani E et al. (2016) Women’s participation in household decision-making and higher dietary diversity: findings from nationally representative data from Ghana. J Health Popul Nutr 35, 1–8. 27245827 10.1186/s41043-016-0053-1 PMC5026004

[28] Kunto YS & Bras H (2018) Women’s empowerment and gender inequality in adolescent nutritional status: evidence from the Indonesian family life survey. J Biosoc Sci 50, 640–665. 29168440 10.1017/S0021932017000566

[29] Tsiboe F , Zereyesus YA , Popp JS et al. (2018) The effect of women’s empowerment in agriculture on household nutrition and food poverty in northern Ghana. Soc Indic Res 138, 89–108.

[30] Carlson GJ , Kordas K & Murray-Kolb LE (2015) Associations between women’s autonomy and child nutritional status: a review of the literature. Matern Child Nutr 11, 452–482. 24521434 10.1111/mcn.12113 PMC6860340

[31] Annette P-Ü , Robert B , Fiona G et al. (2008) Safer Water, Better Health: Costs, Benefits and Sustainability of Interventions to Protect and Promote Health. Geneva: World Health Organization.

[32] Pelto GH , Urgello J , Allen LH et al. (1991) Household size, food intake and anthropometric status of school-age children in a highland Mexican area. Soc Sci Med 33, 1135–1140. 1767283 10.1016/0277-9536(91)90229-6

[33] United Nations Children’s Fund (1991) Strategy for improved nutrition of children and women in developing countries. Indian J Pediatr 58, 13–24. 1937618 10.1007/BF02810402

[34] Christian P & Smith ER (2018) Adolescent undernutrition: global burden, physiology, and nutritional risks. Ann Nutr Metab 72, 316–328. 29730657 10.1159/000488865

[35] Aryeetey R , Lartey A , Marquis GS et al. (2017) Prevalence and predictors of overweight and obesity among school-aged children in urban Ghana. BMC Obes 4, 1–8. 29214030 10.1186/s40608-017-0174-0 PMC5715494

[36] Gyamfi D , Obirikorang C , Acheampong E et al. (2019) Weight management among school-aged children and adolescents: a quantitative assessment in a Ghanaian municipality. BMC Pediatr 19, 1–10. 31651289 10.1186/s12887-019-1772-4 PMC6813048

[37] Ghana Statistical Service (2014) Ghana Living Standards Survey: Round 6 (GLSS6) Main Report. Accra, Ghana: Ghana Statistical Service.

[38] Andam KS , Tschirley D , Asante SB et al. (2018) The transformation of urban food systems in Ghana: findings from inventories of processed products. Outlook Agric 47, 233–243.

[39] Ecker O & Fang P (2016) Economic development and nutrition transition in Ghana: taking stock of food consumption patterns and trends. In Achieving a Nutrition Revolution for Africa: The Road to Healthier Diets and Optimal Nutrition, pp. 28–50 [ N Covic and SL Hendriks , editors]. Washington, DC, USA: International Food Policy Research Institute (IFPRI).

[40] DHS (2006) Guide to DHS statistics. In Demographic and Health Surveys Methodology, pp. 1–147 [ SO Rutstein and G Rojas , editors]. Calverton, Maryland, USA: Demographic and Health Surveys, ORC Macro.

[41] The DHS Programme (2017) The DHS Program – Datasets Account Home. https://dhsprogram.com/data/dataset_admin/index.cfm (accessed September 2018).

[42] De Onis M , Onyango AW , Borghi E et al. (2007) Development of a WHO growth reference for school-aged children and adolescents. Bull World Health Organ 85, 812–819. 18026621 10.2471/BLT.07.043497 PMC2636412

[43] WHO (2011) Haemoglobin Concentrations for the Diagnosis of Anaemia and Assessment of Severity. Geneva, Switzerland: WHO.

[44] Amugsi DA , Mittelmark MB , Lartey A et al. (2014) Influence of childcare practices on nutritional status of Ghanaian children: a regression analysis of the Ghana demographic and health surveys. BMJ Open 4, 1–9. 10.1136/bmjopen-2014-005340 PMC4225227 25366675

[45] WHO & UNICEF (2006) Core Questions on Drinking-Water and Sanitation for Household Surveys. Geneva, Switzerland: World Health Organization.

[46] Reddy VB , Kusuma YS , Pandav CS et al. (2017) Water and sanitation hygiene practices for under-five children among households of Sugali tribe of Chittoor district, Andhra Pradesh, India. J Environ Public Health 2017, 1–7. 10.1155/2017/7517414 PMC5470013 28642797

[47] Bryant K , Anhalt J , Dar B et al. (2014) Establishing a baseline for water, sanitation and hygiene knowledge, attitudes, and practices in rural Ethiopia. J Glob Health 4, 1–11.

[48] Owusu K & Waylen P (2009) Trends in spatio-temporal variability in annual rainfall in Ghana (1951–2000). Weather 64, 115–120.

[49] Berglund PA (2014) Analysis of Survey Data Using the SAS SURVEY Procedures: A Primer. Ann Arbor, MI, USA: Institute for Social Research, University of Michigan, Wisconsin and Illinois SAS User’s Group.

[50] Kassebaum NJ , Jasrasaria R , Naghavi M et al. (2015) A systematic analysis of global anemia burden from 1990 to 2010. Blood J 123, 615–625. 10.1182/blood-2013-06-508325 PMC3907750 24297872

[51] Zimmermann MB , Chaouki N & Hurrell RF (2005) Iron deficiency due to consumption of a habitual diet low in bioavailable iron: a longitudinal cohort study in Moroccan children. Am J Clin Nutr 81, 115–121. 15640469 10.1093/ajcn/81.1.115

[52] Ackah CG , Bortei-Dorku E , Aryeetey E et al. (2009) Global Financial Crisis Discussion Series Paper 5: Ghana. London: Overseas Development Institute.

[53] Thorne-Lyman AL , Valpiani N , Sun K et al. (2010) Dietary diversity and food expenditures are closely linked in Rural Bangladesh, increasing the risk of malnutrition due to the financial crisis. J Nutr 140, 182S–188S. 19923385 10.3945/jn.109.110809

[54] Wegmüller R , Bentil H , Wirth JP et al. (2020) Anemia, micronutrient deficiencies, malaria, hemoglobinopathies and malnutrition in young children and non-pregnant women in Ghana: findings from a national survey. PLoS One 15, 1–19. 10.1371/journal.pone.0228258 PMC6991996 31999737

[55] University of Ghana, GroundWork, University of Wisconsin-Madison et al. (2017) Ghana Micronutrient Survey 2017 (GMS 2017) Final Report. Accra, Ghana: University of Ghana, GroundWork, University of Wisconsin-Madison, KEMRI-Wellcome Trust and UNICEF.

[56] Petry N , Wirth JP , Adu-Afarwuah S et al. (2021) Risk factors for anaemia among Ghanaian women and children vary by population group and climate zone. Matern Child Nutr 17, 1–10. 10.1111/mcn.13076 PMC7988882 32945623

[57] Black RE , Victora CG , Walker SP et al. (2013) Maternal and child undernutrition and overweight in low-income and middle-income countries. Lancet 382, 427–451. 23746772 10.1016/S0140-6736(13)60937-X

[58] Cooke E , Hague S & McKay A (2016) The Ghana Poverty and Inequality Report – 2016: Using the 6th Ghana Living Standards Survey. Brighton, UK: University of Sussex.

[59] Candler T , Costa S , Heys M et al. (2017) Prevalence of thinness in adolescent girls in low- and middle-income countries and associations with wealth, food security, and inequality. J Adolesc Health 60, 447.e1–454.e1. 28110865 10.1016/j.jadohealth.2016.11.003

[60] World Food Programme (2012) Comprehensive Food Security & Vulnerability Analysis. Focus on Northern Ghana. Rome, Italy: Republic of Ghana; WFP.

[61] Ofori-Asenso R , Agyeman AA , Laar A et al. (2016) Overweight and obesity epidemic in Ghana – a systematic review and meta-analysis. BMC Public Health 16, 18. 27938360 10.1186/s12889-016-3901-4 PMC5148846

[62] Moubarac J , Martins APB , Martins B et al. (2012) Consumption of ultra-processed foods and likely impact on human health. Evidence from Canada. Public Health Nutr 14, 5–13. 10.1017/S1368980012005009 PMC10271334 23171687

[63] Popkin BM , Corvalan C & Grummer-Strawn LM (2020) Dynamics of the double burden of malnutrition and the changing nutrition reality. Lancet 395, 65–74. 31852602 10.1016/S0140-6736(19)32497-3 PMC7179702

[64] Buxton CNA (2014) Ghanaian junior high school adolescent’s dietary practices and food preferences: implications for public health concern. J Nutr Food Sci 4, 1–9.

[65] Afrifa–Anane E , Agyemang C , Nii S et al. (2015) The association of physical activity, body mass index and the blood pressure levels among urban poor youth in Accra, Ghana. BMC Public Health 15, 1–9. 25881047 10.1186/s12889-015-1546-3 PMC4376361

[66] Darin-Mattsson A , Fors S & Kåreholt I (2017) Different indicators of socioeconomic status and their relative importance as determinants of health in old age. Int J Equity Health 16, 1–11. 28950875 10.1186/s12939-017-0670-3 PMC5615765

[67] Mirowsky J & Ross CE (2005) Education, cumulative advantage, and health. Ageing Int 30, 27–62.

[68] Mistry SK & Puthussery S (2014) Risk factors of overweight and obesity in childhood and adolescence in South Asian countries: a systematic review of the evidence. Public Health 129, 200–209. 10.1016/j.puhe.2014.12.004 25746156

[69] Teni M , Shiferaw S & Asefa F (2017) Anemia and its relationship with academic performance among adolescent school girls in Kebena District, Southwest Ethiopia. Biotech Health Sci 4, 8.

[70] Assefa S , Mossie A & Hamza L (2014) Prevalence and severity of anemia among school children in Jimma Town, Southwest Ethiopia. BMC Hematol 14, 1–9. 10.1186/2052-1839-14-3 PMC3896819 24433408

[71] Nairz M & Weiss G (2020) Iron in infection and immunity. Mol Aspects Med 75, 1–18. 10.1016/j.mam.2020.100864 32461004

[72] Rah JH , Christian P , Shamim AA et al. (2009) Predictors of stunting and thinness in post-menarcheal adolescent girls in rural Bangladesh. Public Health Nutr 12, 2400–2409. 19344542 10.1017/S1368980009005345

[73] Keino S , Plasqui G , Ettyang G et al. (2014) Determinants of stunting and overweight among young children and adolescents in Sub-Saharan Africa. Food Nutr Bull 35, 167–178. 25076764 10.1177/156482651403500203

[74] Leslie TF & Pawloski LR (2010) Sociodemographic determinants of growth among Malian adolescent females. Am J Hum Biol 22, 285–290. 19693960 10.1002/ajhb.20980

[75] Pawloski LR (2002) Growth and development of adolescent girls from the Segou region of Mali (West Africa). Am J Phys Anthropol 117, 364–372. 11920372 10.1002/ajpa.10037

[76] Riley AP (1994) Determinants of adolescent fertility and its consequences for maternal health, with special reference to rural Bangladesh. Ann N Y Acad Sci 709, 86–100. 8154737 10.1111/j.1749-6632.1994.tb30390.x

[77] De Groot R , Kuunyem MY & Palermo T (2018) Child marriage and associated outcomes in northern Ghana: a cross-sectional study. BMC Public Health 18, 1–12. 10.1186/s12889-018-5166-6 PMC5827991 29482546

[78] Vioque J , Torres A & Quiles J (2000) Time spent watching television, sleep duration and obesity in adults living in Valencia, Spain. Int J Obes 24, 1683–1688. 10.1038/sj.ijo.0801434 11126224

[79] Das GR , Haider SS , Sutradhar I et al. (2019) Association of frequency of television watching with overweight and obesity among women of reproductive age in India: evidence from a nationally representative study. PLoS One 14, 1–13. 10.1371/journal.pone.0221758 PMC6715273 31465465

[80] Ahmed M , Seid A & Kemal A (2020) Does the frequency of watching television matters on overweight and obesity among reproductive age women in Ethiopia? J Obes 2020, 1–7. 10.1155/2020/9173075 PMC7441419 32850148

[81] Bezu BS & Holden S (2014) Land Access and Youth Livelihood Opportunities in Southern Ethiopia: Summary Report. Nairobi: UN-Habitant.

[82] Gbedemah C , Jones N & Pereznieto P (2010) Gendered Risks, Poverty and Vulnerability in Ghana: Is the LEAP Cash Transfer Programme Making a Difference? London, UK: ODI Project Briefing.

[83] Lanerolle-Dias AD , Lanerolle P , Arambepola C et al. (2012) Micronutrient status of female adolescent school dropouts. Ceylon Med J 57, 74–78. 22772785 10.4038/cmj.v57i1.4199

[84] Yamanaka M & Ashworth A (2002) Differential workloads of boys and girls in rural Nepal and their association with growth. Am J Hum Biol 14, 356–363. 12001093 10.1002/ajhb.10030

[85] Hawkes C , Ruel MT , Salm L et al. (2020) Double-duty actions: seizing programme and policy opportunities to address malnutrition in all its forms. Lancet 395, 142–155. 31852603 10.1016/S0140-6736(19)32506-1

[86] Ghana Health Service, Ghana Education Service, UNICEF-Ghana et al. (2019) The Girls’ Iron-Folic Acid Tablet Supplementation (GIFTS) Programme: An Integrated School-Based Nutrition and Health Intervention. Baseline and Follow-On Impact Evaluation in Northern and Volta Regions, Republic of Ghana, 2017–2018. Accra, Ghana: UNICEF-Ghana.

[87] Dubik SD , Amegah KE , Alhassan A et al. (2019) Compliance with weekly iron and folic acid supplementation and its associated factors among adolescent girls in Tamale metropolis of Ghana. J Nutr Metab 2019, 1–12. 10.1155/2019/8242896 PMC6927017 31885910

[88] Gosdin L , Sharma AJ , Tripp K et al. (2020) Barriers and facilitators of iron and folic acid supplementation within a school-based integrated nutrition and health promotion program among Ghanaian adolescent girls. Curr Dev Nutr 4, 1–11. 32914043 10.1093/cdn/nzaa135 PMC7467268

[89] Dewi NU , Nurulfuadi , Aiman U et al. (2020) Food insecurity and anthropometry in adolescents: a literature review. Open Access Maced J Med Sci 8, 234–240.

