# Supplementary material for: Trends and factors associated with the nutritional status of adolescent girls in Ghana: a secondary analysis of the 2003–2014 Ghana demographic and health survey (GDHS) data
Source: Public Health Nutr. 2021 Sep 6;25(7):1912–27. doi: 10.1017/S1368980021003827 (PMC9991666; doi:10.1017/S1368980021003827)
Supplement: Supplementary file 1 [file S1368980021003827sup001.zip › S1368980021003827sup001/S1368980021003827sup001.docx]

**Table S5: Prevalence Difference in Stunting, Thinness, Overweight and Anaemia Among Non-Pregnant Adolescent Girls between 2003-2014: Analysis of the Ghana Demographic Health Survey (GDHS) Data**

| **Malnutrition** | **Crude Estimate** | | | **Adjusted Estimate^1^** | | |
| --- | --- | --- | --- | --- | --- | --- |
|  | **Estimate (%)** | **95%. C.I** | **P-value** | **Estimate (%)** | **95%. C.I** | **P-value** |
| **Stunting** |  |  |  |  |  |  |
| 2003 survey (Ref.) | 0.00 |  |  |  |  |  |
| 2008 survey | -1.55 | -3.92, 0.81 | 0.20 | -1.44 | -3.80, 0.92 | 0.23 |
| 2014 survey | -1.73 | -4.24, 0.77 | 0.17 | -1.45 | -4.00, 1.10 | 0.26 |
| **Thinness** |  |  |  |  |  |  |
| 2003 survey (Ref.) | 0.00 |  |  |  |  |  |
| 2008 survey | -0.39 | -1.71, 0.93 | 0.56 | -0.20 | -1.52, 1.13 | 0.77 |
| 2014 survey | -0.36 | -1.73, 1.02 | 0.61 | -0.59 | -1.98, 0.80 | 0.40 |
| **Overweight** |  |  |  |  |  |  |
| 2003 survey (Ref.) | 0.00 |  |  |  |  |  |
| 2008 survey | 2.15 | -1.04, 5.34 | 0.19 | 3.29 | 0.22, 6.35 | 0.04 |
| 2014 survey | 1.77 | -1.74, 5.28 | 0.32 | 4.29 | 0.74, 7.84 | 0.02 |
| **Anaemia** |  |  |  |  |  |  |
| 2003 survey (Ref.) | 0.00 |  |  |  |  |  |
| 2008 survey | 17.80 | 13.49, 22.10 | <0.0001 | 18.11 | 13.83, 22.39 | <0.0001 |
| 2014 survey | 2.97 | -1.66, 7.61 | 0.21 | 2.82 | -1.76, 7.41 | 0.23 |

^1^Estimates were adjusted for predictors which were significantly associated with the outcome in the pooled analysis
