# Supplementary material for: Trends and factors associated with the nutritional status of adolescent girls in Ghana: a secondary analysis of the 2003–2014 Ghana demographic and health survey (GDHS) data
Source: Public Health Nutr. 2021 Sep 6;25(7):1912–27. doi: 10.1017/S1368980021003827 (PMC9991666; doi:10.1017/S1368980021003827)
Supplement: Supplementary file 1 [file S1368980021003827sup001.zip › S1368980021003827sup001/S1368980021003827sup002.docx]

**Table S4a: Multivariate Predictors of Height-For-Age Z-Score (HAZ) Status Among Non-Pregnant Adolescent Girls: Analysis of the 2003-2014 Ghana Demographic Health Survey (GDHS) Data**

| **Variables** | **2003 (n=983)** | | | **2008 (n=955)** | | | **2014 (n=857)** | | | **Pooled (n=2795)** | | |
| --- | --- | --- | --- | --- | --- | --- | --- | --- | --- | --- | --- | --- |
|  | **Estimate (β)** | **S.E (β)** | ***P-value*** | **Estimate (β)** | **S.E (β)** | ***P-value*** | **Estimate (β)** | **S.E (β)** | ***P-value*** | **Estimate (β)** | **S.E (β)** | ***P-value*** |
| Age |  |  |  |  |  |  | -0.05 | 0.03 | 0.04 |  |  |  |
| Highest educational level of girl |  |  | 0.02 |  |  | 0.002 |  |  | 0.01 |  |  | <0.0001 |
| No education | -0.13 | 0.11 | 0.23 | -0.35 | 0.12 | 0.005 | -0.38 | 0.16 | 0.02 | -0.26 | 0.07 | 0.0003 |
| Primary school | -0.24 | 0.09 | 0.01 | -0.21 | 0.08 | 0.01 | -0.23 | 0.09 | 0.01 | -0.21 | 0.05 | <0.0001 |
| Secondary education /Higher (Ref.) | 0 .00 |  |  | 0 .00 |  |  | 0 .00 |  |  |  |  |  |
| Visited health facility in the last 12 months |  |  |  |  |  |  |  |  |  |  |  |  |
| Yes |  |  |  | 0.17 | 0.07 | 0.02 |  |  |  |  |  |  |
| No (Ref.) |  |  |  | 0 .00 |  |  |  |  |  |  |  |  |
| Relation of girl to the household head |  |  |  |  |  | 0.009 |  |  |  |  |  |  |
| Household head |  |  |  | 0.29 | 0.14 | 0.04 |  |  |  |  |  |  |
| Wife |  |  |  | 0.12 | 0.16 | 0.46 |  |  |  |  |  |  |
| Grand-daughter |  |  |  | 0.00 | 0.11 | 0.99 |  |  |  |  |  |  |
| Other family relation |  |  |  | -0.05 | 0.08 | 0.48 |  |  |  |  |  |  |
| Non-family relation |  |  |  | -0.33 | 0.13 | 0.01 |  |  |  |  |  |  |
| Daughter (ref) |  |  |  | 0 .00 |  |  |  |  |  |  |  |  |
| Household size | -0.02 | 0.01 | 0.03 |  |  |  |  |  |  |  |  |  |
| Household owns land |  |  |  |  |  |  |  |  |  |  |  |  |
| Yes |  |  |  | -0.16 | 0.06 | 0.01 |  |  |  |  |  |  |
| No (Ref.) |  |  |  | 0 .00 |  |  |  |  |  |  |  |  |
| Household wealth index |  |  | 0.03 |  |  |  |  |  | 0.11 |  |  | 0.001 |
| Poorest | -0.09 | 0.11 | 0.41 |  |  |  | -0.18 | 0.10 | 0.12 | -0.21 | 0.07 | 0.002 |
| Poorer | -0.31 | 0.10 | 0.002 |  |  |  | -0.27 | 0.11 | 0.02 | -0.24 | 0.06 | <0.0001 |
| Middle | -0.18 | 0.09 | 0.06 |  |  |  | -0.22 | 0.12 | 0.06 | -0.19 | 0.06 | 0.001 |
| Richer | -0.24 | 0.09 | 0.01 |  |  |  | -0.07 | 0.12 | 0.60 | -0.12 | 0.06 | 0.03 |
| Richest (Ref.) | 0 .00 |  |  |  |  |  | 0 .00 |  |  | 0 .00 |  |  |
| Agro-ecological zone |  |  |  |  |  | 0.093 |  |  | 0.10 |  |  | 0.02 |
| Coastal savannah |  |  |  | -0.01 | 0.07 | 0.95 | 0.08 | 0.08 | 0.32 | -0.02 | 0.04 | 0.69 |
| Guinea/Sudan savannah |  |  |  | 0.17 | 0.09 | 0.043 | 0.21 | 0.10 | 0.03 | 0.15 | 0.06 | 0.01 |
| Forest |  |  |  |  |  |  |  |  |  | 0 .00 |  |  |
| Year of survey |  |  |  |  |  |  |  |  |  |  |  | 0.05 |
| 2003 (Ref.) |  |  |  |  |  |  |  |  |  | 0 .00 |  |  |
| 2008 |  |  |  |  |  |  |  |  |  | 0.01 | 0.04 | 0.85 |
| 2014 |  |  |  |  |  |  |  |  |  | 0.10 | 0.05 | 0.03 |
| **Model Fit Statistics** |  |  |  |  |  |  |  |  |  |  |  |  |
| R-Square |  |  | 0.03 |  |  | 0.05 |  |  | 0.04 |  |  | 0.03 |
| Root MSE |  |  | 0.91 |  |  | 0.89 |  |  | 0.86 |  |  | 0.89 |
| Model (F-Value) |  |  | 4.93 |  |  | 4.84 |  |  | 2.78 |  |  | 6.64 |
|  |  |  | <0.0001 |  |  | <0.0001 |  |  | 0.004 |  |  | <.0001 |

β: regression coefficient; SE (β): standard error of regression coefficient; ref: reference group; MSE: mean square of residuals

**Table S4b: Multivariate Predictors of Body-Mass-Index (BMI)-For-Age Z-Score (BAZ) Status Among Non-Pregnant Adolescent Girls: Analysis of the 2003-2014 Ghana Demographic Health Survey (GDHS) Data**

| **Variables** | **2003 (n=983)** | | | **2008 (n=955)** | | | **2014 (n=857)** | | | **Pooled (n=2795)** | | |
| --- | --- | --- | --- | --- | --- | --- | --- | --- | --- | --- | --- | --- |
|  | **Estimate (β)** | **S.E (β)** | ***P-value*** | **Estimate (β)** | **S.E (β)** | ***P-value*** | **Estimate (β)** | **S.E (β)** | ***P-value*** | **Estimate (β)** | **S.E (β)** | ***P-value*** |
| Age |  |  |  | 0.07 | 0.03 | 0.01 |  |  |  |  |  |  |
| Frequency of listening to the radio in the past week |  |  |  |  |  |  |  |  |  | 0.04 | 0.01 | 0.01 |
| Frequency of watching television in the past week | 0.09 | 0.03 | 0.001 |  |  |  |  |  |  |  |  |  |
| Autonomy |  |  |  |  |  |  | 0.06 | 0.03 | 0.04 |  |  |  |
| WASH index |  |  |  |  |  |  | 0.09 | 0.04 | 0.01 |  |  |  |
| Household wealth index |  |  | 0.02 |  |  | 0.001 |  |  | 0.006 |  |  | <0.0001 |
| Poorest | -0.28 | 0.10 | 0.005 | -0.36 | 0.11 | 0.001 | -0.32 | 0.11 | 0.004 | -0.44 | 0.06 | <0.0001 |
| Poorer | -0.26 | 0.10 | 0.01 | -0.41 | 0.12 | 0.001 | -0.39 | 0.11 | 0.001 | -0.43 | 0.06 | <0.0001 |
| Middle | -0.29 | 0.10 | 0.003 | -0.26 | 0.10 | 0.01 | -0.10 | 0.11 | 0.38 | -0.26 | 0.06 | <0.0001 |
| Richer | 0.02 | 0.09 | 0.80 | -0.09 | 0.10 | 0.40 | -0.15 | 0.13 | 0.26 | -0.11 | 0.06 | 0.08 |
| Richest (Ref.) | 0.00 |  |  |  |  |  | 0.00 |  |  |  |  |  |
| Agro-ecological zone |  |  | 0.004 |  |  |  |  |  |  |  |  |  |
| Coastal savannah | 0.02 | 0.07 | 0.78 |  |  |  |  |  |  |  |  |  |
| Guinea/Sudan savannah | -0.27 | 0.08 | 0.001 |  |  |  |  |  |  |  |  |  |
| Forest (ref.) | 0 .00 |  |  |  |  |  |  |  |  |  |  |  |
| Year of survey |  |  |  |  |  |  |  |  |  |  |  | 0.001 |
| 2003 (Ref.) |  |  |  |  |  |  |  |  |  | 0 .00 |  |  |
| 2008 |  |  |  |  |  |  |  |  |  | 0.04 | 0.05 | 0.42 |
| 2014 |  |  |  |  |  |  |  |  |  | 0.18 | 0.05 | 0.0001 |
| **Model Fit Statistics** |  |  |  |  |  |  |  |  |  |  |  |  |
| R-Square |  | 0.09 |  |  |  | 0.04 |  | 0.07 |  |  | 0.05 |  |
| Root MSE |  | 0.85 |  |  |  | 0.92 |  | 0.85 |  |  | 0.88 |  |
| Model (F-Value) |  | 12.88 | <0.0001 |  | 5.85 | <0.0001 |  | 5.85 | <0.0001 |  | 13.57 | <0.0001 |

β: regression coefficient; SE (β): standard error of regression coefficient; ref: reference group; MSE: mean square of residuals; WASH, Household water and sanitation

**Table S4c: Multivariate Predictors of Haemoglobin (Hb) status Among Non-Pregnant Adolescent Girls: Analysis of the 2003-2014 Ghana Demographic Health Survey (GDHS) Data**

| **Variables** | **2003 (n=983)** | | | **2008 (n=955)** | | | **2014 (n=857)** | | | **Pooled (n=2795)** | | |
| --- | --- | --- | --- | --- | --- | --- | --- | --- | --- | --- | --- | --- |
|  | **Estimate (β)** | **S.E (β)** | ***P-value*** | **Estimate (β)** | **S.E (β)** | ***P-value*** | **Estimate (β)** | **S.E (β)** | ***P-value*** | **Estimate (β)** | **S.E (β)** | ***P-value*** |
| Girl has ever given birth |  |  |  |  |  |  |  |  |  |  |  |  |
| Yes | -2.97 | 1.09 | 0.01 |  |  |  |  |  |  |  |  |  |
| No (Ref.) | 0.00 |  |  |  |  |  |  |  |  |  |  |  |
| Religion |  |  | 0.001 |  |  |  |  |  |  |  |  |  |
| Christian | 3.78 | 1.17 | 0.001 |  |  |  |  |  |  |  |  |  |
| Muslim | 1.56 | 1.26 | 0.22 |  |  |  |  |  |  |  |  |  |
| Other (Ref.) | 0.00 |  |  |  |  |  |  |  |  |  |  |  |
| Relation of girl to the household head |  |  |  |  |  | 0.15 |  |  |  |  |  |  |
| Household head |  |  |  | 3.81 | 2.60 | 0.14 |  |  |  |  |  |  |
| Wife |  |  |  | 0.93 | 2.27 | 0.68 |  |  |  |  |  |  |
| Grand-daughter |  |  |  | 1.70 | 1.95 | 0.38 |  |  |  |  |  |  |
| Other family relation |  |  |  | 2.16 | 1.50 | 0.15 |  |  |  |  |  |  |
| Non-family relation (Ref.) |  |  |  | 5.35 | 2.37 | 0.02 |  |  |  |  |  |  |
| Daughter (Ref.) |  |  |  |  |  |  |  |  |  |  |  |  |
| Never married (Ref.) |  |  |  | 0.00 |  |  |  |  |  |  |  |  |
| Nutritional status |  |  | 0.03 |  |  | 0.05 |  |  |  |  |  | 0.02 |
| Underweight | -4.92 | 1.83 | 0.0 | -7.82 | 3.95 | 0.049 |  |  |  | -6.80 | 2.73 | 0.01 |
| Overweight/Obese | -0.44 | 1.33 | 0.74 | 2.82 | 2.11 | 0.18 |  |  |  | 1.37 | 0.97 | 0.16 |
| Normal weight (Ref.) | 0.00 |  |  | 0.00 |  |  |  |  |  | 0.00 |  |  |
| Household wealth index |  |  |  |  |  |  |  |  | 0.01 |  |  |  |
| Poorest |  |  |  |  |  |  | 2.72 | 1.85 | 0.14 |  |  |  |
| Poorer |  |  |  |  |  |  | -1.67 | 2.10 | 0.43 |  |  |  |
| Middle |  |  |  |  |  |  | 3.86 | 1.87 | 0.04 |  |  |  |
| Richer |  |  |  |  |  |  | 4.21 | 1.84 | 0.02 |  |  |  |
| Richest (Ref.) |  |  |  |  |  |  | Ref. |  |  |  |  |  |
| Agro-ecological zone |  |  | 0.003 |  |  | 0.001 |  |  |  |  |  |  |
| Coastal savannah | --3.08 | 0.91 | 0.001 | 4.03 | 1.33 | 0.003 |  |  |  |  |  |  |
| Guinea/Sudan savannah | -0.55 | 0.71 | 0.44 | 4.90 | 1.48 | 0.001 |  |  |  |  |  |  |
| Forest (Ref.) | 0.00 |  |  | 0.00 |  |  |  |  |  |  |  |  |
| Year of survey |  |  |  |  |  |  |  |  |  |  |  |  |
| 2003(Ref.) |  |  |  |  |  |  |  |  |  |  |  | <0.0001 |
| 2008 |  |  |  |  |  |  |  |  |  | -7.73 | 0.71 | <0.0001 |
| 2014 |  |  |  |  |  |  |  |  |  | -2.57 | 0.71 | 0.0003 |
| **Model Fit Statistics** |  |  |  |  |  |  |  |  |  |  |  |  |
| R-Square |  | 0.02 |  |  | 0.04 |  |  | 0.03 |  |  | 0.05 |  |
| Root MSE |  | 14.81 |  |  | 15.73 |  |  | 13.70 |  |  | 14.90 |  |
| F-Value |  | 7.22 | <0.0001 |  | 3.70 | <0.0001 |  | 3.61 | 0.007 |  | 32.57 | <0.0001 |

β: regression coefficient; SE (β): standard error of regression coefficient; ref: reference group; MSE: mean square of residuals
