# Supplementary material for: Trends and factors associated with the nutritional status of adolescent girls in Ghana: a secondary analysis of the 2003–2014 Ghana demographic and health survey (GDHS) data
Source: Public Health Nutr. 2021 Sep 6;25(7):1912–27. doi: 10.1017/S1368980021003827 (PMC9991666; doi:10.1017/S1368980021003827)
Supplement: Supplementary file 1 [file S1368980021003827sup001.zip › S1368980021003827sup001/S1368980021003827sup003.docx]

**Table S1: Flow Chart in the Population for Analysis**

|  | **Year of Survey** | | | | |
| --- | --- | --- | --- | --- | --- |
|  | **1993** | **1998** | **2003** | **2008** | **2014** |
| Number of women in fertile age (15-49 years) | 4562 | 4843 | 5691 | 4916 | 9396 |
| Number of Adolescents (15-19 years) | 140 | 98 | 1046 | 1004 | 887 |
| Number of those with anthropometry and/or haemoglobin data | 140 | 98 | 1046 | 1004 | 887 |
| Number of those flagged for anthropometry z-score | - | - | 19 | 11 | - |
| Remainder | 140 | 98 | 1027 | 993 | 887 |
| Number of those pregnant | 9 | - | 38 | 38 | 30 |
| **Population for analysis (non-pregnant girls)** | **131** | **98** | **955** | **955** | **857** |
| Analysed | No | No | Yes | Yes | Yes |
