# Supplementary material for: Trends and factors associated with the nutritional status of adolescent girls in Ghana: a secondary analysis of the 2003–2014 Ghana demographic and health survey (GDHS) data
Source: Public Health Nutr. 2021 Sep 6;25(7):1912–27. doi: 10.1017/S1368980021003827 (PMC9991666; doi:10.1017/S1368980021003827)
Supplement: Supplementary file 1 [file S1368980021003827sup001.zip › S1368980021003827sup001/S1368980021003827sup004.docx]

**Table S2a: Attitude toward wife-beating index from GDHS data for adolescent girls**

| **Variables** | **Survey Year** | | | | | **Scoring**  **Yes=0**  **No=1** |
| --- | --- | --- | --- | --- | --- | --- |
|  | **1993** | **1998** | **2003** | **2008** | **2013** |  |
| ***Wife beating is justified if :*** |  |  |  |  |  |  |
| 1. *Goes out without telling him* |  |  | √ | √ | √ |  |
| 1. *Neglects the children* |  |  | √ | √ | √ |  |
| 1. *Argues with him* |  |  | √ | √ | √ |  |
| 1. *Refuses to have sex with him* |  |  | √ | √ | √ |  |
| 1. *Burns the food* |  |  | √ | √ | √ |  |
| ***Maximum attainable score for attitude toward wife beating*** | **N/A** | **N/A** | **5** | **5** | **5** |  |

N/A, Not applicable

**Table S2b: Scoring format for household water and sanitation (WASH) index**

| No | Variable | Score |
| --- | --- | --- |
| 1 | **Household water index (WI): score range 0-2** |  |
| 1.1 | Improved source of drinking |  |
|  | - Piped water supply into the dwelling - Piped water to a yard/plot - Public tap/standpipe - Tube well/borehole - Protected dug well - Protected spring - Rainwater - If the source of drinking water is packaged/sachet-water or bottled and source of non-drinking water is improved | 1 |
|  | - Any other water source | 0 |
| 1.2 | Round-trip time in water haulage between household and water source |  |
|  | - If the time spent ≤ 30 minutes | 1 |
|  | - If time spent > 30 minutes | 0 |
| 2 | **Household sanitation index (HSI): score range 0-1** |  |
| 2.1 | Improved household sanitation facility using the type of toilet |  |
|  | - Flush/pour-flush toilet or latrine that flushes to a sewer, septic tank or pit. - Ventilated improved pit (VIP) latrine - Pit latrines with the pit well covered by a slab - Composting toilets are also considered improved | 1 |
|  | - Any other toilet | 0 |
|  | **The maximum attainable score for household water and sanitation (WASH)** | **3** |
