# Supplementary material for: Trends and factors associated with the nutritional status of adolescent girls in Ghana: a secondary analysis of the 2003–2014 Ghana demographic and health survey (GDHS) data
Source: Public Health Nutr. 2021 Sep 6;25(7):1912–27. doi: 10.1017/S1368980021003827 (PMC9991666; doi:10.1017/S1368980021003827)
Supplement: Supplementary file 1 [file S1368980021003827sup001.zip › S1368980021003827sup001/S1368980021003827sup005.docx]

**Table S3: Prevalence rates and 95% confidence intervals of nutritional status indicators of non-pregnant adolescent girls from the 2003-2010 GDHS**

| **Nutritional status** | **Prevalence (95% C.I)** | | |
| --- | --- | --- | --- |
|  | **2003 survey** | **2008 survey** | **2014 survey** |
| Stunting | 7.9 (6.4, 9.5) | 6.4 (4.7, 8.0) | 6.2 (4.4, 8.0) |
| Thinness | 2.0 (1.1, 2.8) | 1.6 (0.7, 2.4) | 1.6 (0.7, 2.5) |
| Overweight/obesity | 10.0 (7.8, 12.2) | 12.2 (10.0, 14.3) | 11.8 (9.2, 14.3) |
| Anaemia | 44.3 (40.7, 47.9) | 62.1 (58.5, 65.6) ^a^ | 47.3 (43.3, 51.2) |

^a^ Statistically significantly higher than the 2003 survey with alpha 0.05
